# The Middlesex Hospital

**Published:** 1902-06-28

**Authors:** 


### Around the Hospitals.

### THE MIDDLESEX HOSPITAL.

### THE YEAR'S WORK.

In all departments the number of patients treated in the hospital was 3,777, but at least 150 more would have been admitted but for the necessary closing of four of the wards during alterations. There were 45,084 out-patients. The average cost of each in-patient was £2 1s. 3¾d., and of each out-patient 1s. 6¼d. The cost of each occupied bed was £93 11s. 4d. The average stay was, for medical patients, 26·76 days; surgical, 25·14; cancer, 94·88; and gynæcological, for the first time reckoned separately, 29 35.

The total income was £34,650 2s. 1d., and the total expenditure was £39,613 6s. 8d. To the latter £1,000 has to be added in respect of a legacy to the Permanent Endowment Fund, not available for current expenses, giving a deficit of £5,933 4s. 7d., all of which is attributable to building improvements. The deficit for 1900 was £5,092 8s. 5d. There was a rise of £228 1s. 6d. in annual subscriptions, due mainly to Lord Howard de Walden's contribution of £300 a year, but donations have fallen by £1,032 18s. 2d. The Hospital Sunday Fund gave nearly £230 less than in 1900, and £250 had to be returned to King Edward's Fund, owing to the hospital's inability to comply with certain conditions "which would have involved the general funds in a liability greatly exceeding the sum in question." The balance of the donation was £750, £1,000 being the usual amount of the fund's contribution. Dividends and rents have increased.

The extraordinary income amounted to £18,191 6s. 3d., being entirely made up of legacies. Mr. Henry Vaughan left £5,000, Mrs. Henry Harris £2,411 9s. 8d., and the sale of freehold property at Kingston-on-Thames produced £5,660. The legacy of £1,000 bequeathed by Mr. T. Kincaid Hardie to the Permanent Endowment Fund has been specially invested.

The ratio of management expenses to total ordinary expenditure was less than 5 per cent. Nearly £1,000 was spent on renewal of linen, etc., but there was a saving of £100 on the cost of provisions, owing, presumably, to the closing of the four wards. The new photographic department cost £120 8s.

#### New Buildings.

The north-east wing of the hospital has been extended, and the enlarged Helena and Seymour wards, each with 20 beds, have been re-opened. The new building comprises a separate sanitary block. The space formerly occupied by "Hawkins" and "Prudhoe" wards has been converted into new kitchen offices; the cost of the cooking apparatus was £704 7s. A tender for £8,283 for the proposed extension of the nurses' home, on the site of Nos. 19, 21, and 23 Cleveland Street, has been accepted: the street elevation will be a continuation of the Institute and Old Nurses' Home; the garden front will be carried out in red brick and terra-cotta, to harmonise with the Residential College. There will be a new sitting-room, large refectory, and about forty bedrooms; these will enable the committee to make necessary additions to the nursing staff. The pavilion for open-air treatment of tuberculosis at the Convalescent Home, Clacton-on-Sea, is being completed, and the space hitherto set apart for this will soon be available for ordinary cases. The new laundry at Hendon is expected to be ready during the summer, when all the washing will be sent there, to be dealt with under efficient and healthy conditions.

#### The Cancer Fund.

The ordinary income of this fund was slightly less than in 1900, being £3,014 7s. 7d., instead of £3,761 1s. 4d. This is owing to a fall in donations, for subscriptions remain at par, and returns from invested property have risen. The extraordinary income from legacies was £1,411 13s. 2d., of which £1,200 was for the endowment of a bed under the will of Miss Emmett, and Mrs. Blagrove-Paton has endowed a bed (£1,050) in memory of her late husband. The sum of £2,250 has been separately invested, in accordance with the wish of the donors. The total expenditure was £4,915 3s. 5d., which is slightly less than last year's. The cost of administration was £84 14s.

The Cancer Investigation Fund is again a separate account. Donations almost doubled themselves in 1901, but annual subscriptions are still small. The former were £1,418 2s. 3d., and the latter £53 13s. The expenditure was £705 6s. 10d., of which £501 14s. was devoted to the salaries of the director and his assistants, and £203 12s. 10d. to scientific apparatus and appliances; there is a balance in hand of £2,174 2s. 4d. The Cancer Research Laboratories have been in working order for nearly two years, and during the present year a report will be issued, giving a detailed account of the work already done.

